# Past, Present and Future of Membrane Technology in Spain

**DOI:** 10.3390/membranes11110808

**Published:** 2021-10-24

**Authors:** José I. Calvo, Clara Casado-Coterillo, Antonio Hernández

**Affiliations:** 1 Surfaces and Porous Materials (SMAP) Group, Associated Research Unit to CSIC, UVa-innova Bldg, P. Belén, 11 and Institute of Sustainable Processes (ISP), Dr. Mergelina, s/n, University of Valladolid, 47071 Valladolid, Spain; antonio.hernandez@uva.es; 2 Department of Chemical and Biomolecular Engineering, Universidad de Cantabria, Av. Los Castros s/n, 39005 Santander, Spain

**Keywords:** membrane technology, membrane processes, Spanish research groups, bibliometric analysis

## Abstract

The following review aims at analyzing the contribution of Spanish researchers to membrane science and technology, with a historical compilation of the main milestones. We used a bibliometric analysis based on the Scopus database (1960–2020) dealing with 8707 documents covering the different disciplines and subject areas where membranes are involved. Furthermore, the information has been updated to the present moment of writing this manuscript in order to include the latest research lines and the different research groups currently active in Spain, which may lead the way to the development of the field in the coming years.

## 1. Introduction

### 1.1. Early Historic Background

The filtration process can indeed be considered the oldest existing separation technique. It was already known and exploited by civilizations as ancient as the Egyptians and the Chinese. However, limiting ourselves to Europe, we can consider the work of Lucretius (95–55 BC), “De Rerum Natura”, as the beginning of the science of separation in our continent [1].

However, this beginning was quite slow, since more than 15 centuries had to pass in order to find new works related to the subject. Among these works, that of the French Abbé Nollet is often considered a pioneer in membranology. In 1748, Nollet made the first experiment with semi-permeable membranes (porcine bladders, in fact) [2]. The separation studies carried out throughout the nineteenth century, already more numerous and systematic, led to a better understanding of the separation processes and the laws that govern them. Very important in these aspects are the works of Fick on diffusion [3], Traube [4], Pfeffer [5], and Van’t Hoff [6,7] with pressure osmotic, and many other interesting works from Raoult, Graham, etc.

From Traube’s pioneering work, Zsigmondy was the first to develop a cellulose nitrate membrane suitable for the sterilization of small amounts of fluid, [8], later marketed by Sartorius [9].

A really entertaining and interesting insight into these early years of membranology can be found in [10]. Figures such as Abbé Nollet, Dutrochet, Doebereiner, Graham, and many other pioneers of membrane science parade through this pleasant historical “review”. A work more related to membrane technology, although already classic is that of Lonsdale, the first Editor in Chief of the *Journal of Membrane Science* [11].

However, from a practical point of view, the true starting point of what we can without fear call the membrane technology revolution can be found in the asymmetric cellulose acetate membrane developed by Loeb and Sourirajan in 1960 [12], which allowed solving some of the necessary challenges to obtain a material (the membrane) with the selectivity and stability features necessary to be applied on a large scale in desalination of seawater by reverse osmosis.

Before the 1980s different processes with membranes are used industrially and proposed scientifically as promising alternatives for other applications. In the late 70s and 80s, membrane technology was starting to be presented as a strategic science and technology with definitions, history, and agenda [13].

In all these pioneering studies, the contribution of Spanish researchers in membrane technology is practically absent [14,15].

By the end of the 1970s, when membrane technology began to be mature, research in Spain was also ready to make its input. As it occurred in the rest of the world, the problems addressed by membrane processes in southern countries focused on the poor quality of drinking water and water supply [16], as well as on wastewater management from dairy and paper industries [17], and membrane researchers started to formulate this in terms of membrane technology for example by using reverse osmosis. The first Spanish contributions to membrane processes were thus found in general chemical engineering journals [18,19,20] or more specific journals dealing with actual industrial or environmental challenges in food, paper, and metallurgical industries. Thus, it is not surprising to find that the first Spanish contributions to membrane research will be related to the then recently discovered cellulose acetate membranes [21,22,23,24] as well as others dealing with reverse osmosis water desalination plants [25,26]. Two previous articles on ion-exchange membranes could be remarked [27,28] although, apparently without subsequent continuity. Regarding the work of Muro et al. [26] on seawater desalination plants, this is mostly a national desalination policies review and does not present actual research. Likewise, Guzmán’s article (followed by a later one in 1984, [29], are descriptive works on the underlying engineering in two water desalination plants in the Canary Islands. Thus, we can consider the group born at the Complutense University of Madrid, by the hand of Juan Ignacio Mengual, as the first active research group on synthetic membranes in Spain. On his part, Cristóbal Fernández Pineda, upon obtaining his teaching position at the University of Malaga, would create his own group at that university, also very active in these early years of membranology in Spain.

These first names already allow us to note a curious characteristic of membrane research in Spain. Surprisingly most of the research groups on the subject come from departments of Applied Physics of Spanish Universities. So are Mengual and Fernández Pineda, but also Fernando Tejerina and Javier Garrido and Julio Pellicer in the University of Valencia. José Antonio Ibáñez and Antonio Hernández were disciples of Fernando Tejerina in The Autonomous University of Barcelona and afterwards in Murcia and Valladolid, respectively. All of these published works are related to the application of the Thermodynamics of Irreversible Processes on the explanation of the different electrokinetic phenomena observed in charged membranes. 

Thus, these first works dealt with salt diffusion [30], transport models [31], membrane current, membrane potential, charge densities, or ionic permeabilities. In parallel, the study of the properties of permeability [32] and solute separation [33] in membranes would continue its course. In the next years, Fernández-Pineda, very active in publishing, would produce several papers on hydrodynamics and separation properties of cellophane membranes.

A colleague of Fernández-Pineda at the same University (Málaga) as—with whom she also publishes several articles—professor Juana Benavente specialized in electrokinetic properties, firstly using cellophane membranes [34], but later with many other membrane materials [35].

Meanwhile, Antonio Hernández from the University of Valladolid published works on the permeation of ionic salts species through various polymeric membranes [36,37]. Another group of papers, such as Garrido et al., 1989, Díaz et al., 1989, or Alegret et al., 1989 [38,39,40], focused on membranes not in industrial separation but as an important part of electrodes and sensors. 

In 1987, new names (not linked to Applied Physics) began to appear dedicated to membrane research (apart from new articles associated with desalination companies [41]) especially chemical engineers [42], which would give rise to a thriving group of researchers at the Polytechnic University of Valencia, for example.

The end of the 80s registers the appearance of one of the largest membrane groups in Spain, that led by Inmaculada Ortiz, initially at the University of the Basque Country, and since the early 90s, at the University of Cantabria. Being part of a purely Chemical Engineering department, Prof. Ortiz published her first paper on liquid membranes in 1988 in *Industrial and Engineering Chemistry Research* [43]. This group of chemical engineers introduced a strong focus on the design and development of membrane processes using process simulation tools and explored the scale-up of membrane processes with numerous pilot plant case studies in collaboration with the chemical industry.

The young but increasing membrane technology field attracts many researchers due to the big range of applications where synthetic membranes can offer a solution for separation problems in the industry. A paper in 1988, by researchers of the Instituto de la Grasa from the Spanish Superior Research Council, CSIC [44], presented the application of Ultrafiltration to the regeneration of olive brines.

Alonso et al. used membrane filters to control the population of *Pseudomonas aeruginosa* in water [45].

At the University of Granada, there was from the beginning a group active in electrokinetic studies on porous plugs [46,47], which later evolved into a group using network thermodynamics to model membrane transport [48]. More studies continue to evaluate the performance of RO in water desalination [49,50]. 

The group of Organic Chemistry from the Instituto de Ciencia y Tecnología de Polímeros (CSIC) started to publish on the properties of UF membranes cast from polyamide, being one of the first groups in Spain researching on membrane preparation [51,52].

In 1992, the group of Jose Coca, from the University of Oviedo, started to publish regarding the application of reverse osmosis membrane processes to dairy whey treatment [53], a research line which later has been revealed as very productive [54,55].

The mid-90s saw other research groups with different backgrounds starting to contribute to membrane science as an emerging technology. One of those was the group of University of Zaragoza, led by Jesús Santamaría, who published in 1994 their first paper on ceramic membrane reactors [56] and soon became an internationally recognized group on zeolite and other ceramic membranes [57].

Step by step, the 80s and early 90s would see a progressive increase in the applications of membrane processes, and with them, of the publications of Spanish researchers, increasingly associated with groups of chemical engineers or chemists, mostly interested in the different processes in which membranes were finding increasing potential but also in the way to develop new membranes with selected features.

**Figure 1:**
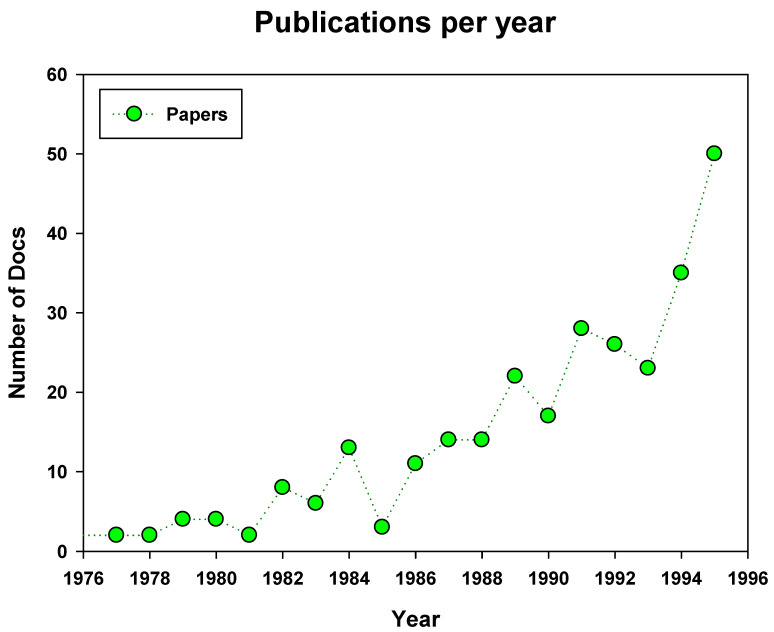
Spanish based membrane publications in international journals from 1970 to 1995.

In fact, from 1990 onwards, there is a clear increase in the number of publications from Spanish researchers related to membrane technology as can be seen in Figure 1. This trend has been maintained and even increased in the following decades, also linked to an important general rise of scientific research in Spain. Several factors contributed to this increase:

(1) University Reform Law, issued in 1983, was the first law in democracy that sought to modernize the obsolete and undemocratic structure of the Spanish university. On the other hand, the opening and democratization of the access to the university in these years led to an almost exponential increase in the number of students, rising from 170,000 in the 1959–60 academic year to more than 1,440,000 in the year 1994–95 [58].
(2) The increase in the number of students led to the creation of new universities and the proportional increase in the number of professors who, in addition, were now required to have a research curriculum to stabilize their positions.
(3) Considering that in Spain, the research work was always closely linked to the University, with little contribution until this century from the industrial sector, such a notable increase in university size and quality, led to a consequent expansion of research in general and research in applied fields such as membrane technology in particular.
(4) In parallel, the 90s would see an important effort from the various democratic governments to provide Spanish researchers with funding. Thus, the consolidated groups and new groups created to cover new emerging topics found more financing possibilities that allowed them to acquire increasingly complex instrumental equipment, take part regularly in internationally recognized congresses and send their best young researchers to foreign research centers establishing collaborations that enriched the research background of the national groups.

**Figure 2:**
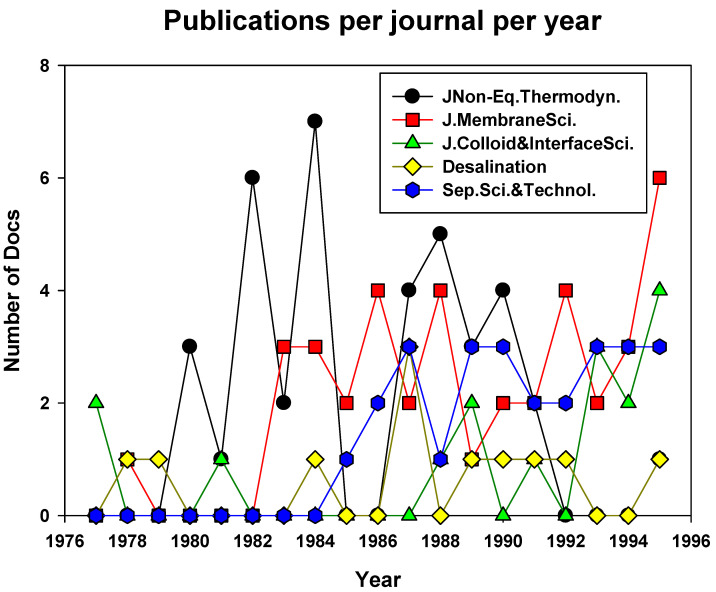
Spanish based membrane-related publications in selected international journals from 1970 to 1995.

This clearly somewhat superficial analysis of the first decades of membrane research in Spain can be completed with a view to the main source of publication of Spanish research reports dealing with membranes. Between 1970 and1995, 38 out of the 286 articles reported were published in the *Journal of Non-equilibrium Thermodynamics*, a journal clearly focused on the thermodynamic analysis of irreversible processes, including electrokinetic processes. The outcome of this journal is closely followed (36 documents) by the main reference on membrane science, the *Journal of Membrane Science*, founded in 1976 “to draw together a new field, tentatively called ‘membranology’, which turned to ‘membrane technology’’’, and has included contributions from Spanish researchers from almost the very beginning [23]. The relative importance of these journals can be seen in Figure 2, where the number of papers in the main international journals during these pioneering years is depicted.

Journals mostly devoted to physical properties of separation of charged species as *Journal of Non-Equilibrium Thermodynamics* or *Journal Colloid and Interface Science*, will be preferred in the initial part of the period while, as more and more researchers move to study the process itself and its applications, the journals mainly devoted to separation as *Desalination*, *Separation Science and Technology* or, the most important one, *Journal of Membrane Science* would start to attract maximum attention to the Spanish membrane researchers. From 1988 onwards, Spanish researchers started contributing to the development of membrane processes in journals focused on chemical engineering and chemical technology, that already included sections devoted to separation such as *Industrial & Engineering Chemistry Research* (7), *AIChE Journal* (4), and *Chemical Engineering Science* (4). However, these latter are not included in Figure 2 for clarification.

The fact that a broader list of journals was available for authors to publish their contributions shows us the rise of applications and approaches related to membranes that Spanish researchers were beginning to explore. Thus, in 1977 or 1978, while the total number of publications in the journals selected in Figure 2 almost equaled 100% of the existing ones, in 1995, only 30% of the total articles were published in those 5 journals in Figure 2, the rest being published elsewhere. They were thus distributed among 27 different journals and magazines, covering a wide variety of fields, from catalysis, materials science to chemical or food engineering.

Another important milestone was the creation of a Spanish Membrane Group (Grupo Español de Membranas, GEM), a consequence of the interest of Spanish researchers who had begun to meet in several reputed international congresses on the topic (Euromembrane or the International Congress on Membranes and Membrane Processes, ICOM, as flag representation, and their specific sub-sections on Inorganic Membranes, ICIM, or Membrane Reactors, ICCMR, among others) and wanted to be aware of mutual research efforts and synergies. Certainly, the birth of the GEM was influenced by the European Society of Membrane Science and Technology (now European Membrane Society, EMS) founded in 1982, and in some way imitated it. The group, founded in the beginning of the 90s, was initially chaired by Juan Ignacio Mengual, with Antonio Hernández as secretary, but the unfortunate loss of its president and the growing drive of chemical engineers would result in the slow languishment of GEM until its effective disappearance. The spirit of association could be then maintained for the duration of the thematic network on selective membranes promoted by Ane Urtiaga and funded by the Spanish Ministry of Science and Innovation in 2002–2004, which fostered the exchange of ideas and the transfer of knowledge on membrane science and technology from the academy to the industry and society.

An important impulse pushed by the research groups actively involved in national associations would translate into the necessity of the creation of some type of international scientific congress that allows Iberian researchers to meet and discuss their common work. This resulted in the series of Ibero-American Congresses on Membrane Science and Technology (CITEM) of which X editions have been held to date. The first one was organized by the Universidad de Murcia in Murcia in 1992. Further editions were held at: Rio de Janeiro, 1994; Aveiro, 2001; Florianópolis, 2003; Valencia, 2005; Campina Grande, 2007; Sintra, 2010; Salta, 2012; Santander 2014 and Mexico DF, 2016. In all cases the CITEM congresses have proved a genuine ability to promote the mutual collaboration between Ibero-American research groups, obviously with more Iberian researchers was major in those celebrated in Spain or Portugal while Ibero-American researchers were the majority in the meetings held at the other side of the Atlantic Sea.

### 1.2. Data Resource and Methodology

The aim of this work is the bibliometric analysis of the research performed in the last century in Spain in the light of how they can address the challenges of the present. Therefore, this paper will be useful to focus the global panorama of the research in membranes with Spain as one of the authors’ affiliation countries.

As commented before, after this slow introduction of membrane technology in the Spanish research, the subject was tackled by many other researchers of various working fields. In order to grasp a preliminary idea of the relevance of Spanish contributions in the scientific literature on membrane technology, we have carried out a non-exhaustive bibliometric analysis search to find the number of papers published in international journals authored by Spanish researchers and technologists. A bibliometric analysis is a systematic evaluation of the records found under specific keywords is used to quantitatively determine the characteristics of the research and provide an overview of the trends in this topic regarding most emerging applications, which has not been the object of similar studies before, as far as we know. The tool used is a bibliometric analysis of the literature indexed in the Scopus database [59] up to 2021. In this search we have used following the marks:

− Keyword: Membrane
− Affiliation country: Spain

In an initial search, this led to a significantly large amount of references, including many devoted to biological membranes or other fields not related with the use of synthetic membranes for separations. We also tried more specific keywords as “Membrane Technology” or Membrane Processes”. However, these searches left out a number of very important publications. For example, using “Membrane Technology” most papers published in the Journal of Membrane Science were not selected. Therefore, we decided to continue using this general keyword but eliminate all references coming from journals specializing in Medicine, Immunology, Therapies or similar subjects. This finally led to a total amount of 13,492 papers, of which the analysis was then limited up to 8707 documents after eliminating those not related to membrane science and technology. For this further reduction, the keywords included by authors were used and also the name of the journals where the papers were published. Therefore, papers containing keywords such as: apoptosis, gene expression, cytology, rats, mice, or similar words, were excluded. Similarly, those papers published in journals as: *Physiologia Plantarum*, *Journal of Experimental Botany*, *Theoretical Computer Science* or *Neuroscience Letters*, among others, were also discarded. 

Obviously, in this selection, there still remain many papers not really related to what we usually consider membrane technology while surely a large number of interesting papers is also missing. Yet, this selection serves to grasp a quite approximative idea about the membrane scientific production and evolution in Spain (and certainly not only in Spain, as many of the papers included are international collaborations). 

**Figure 3:**
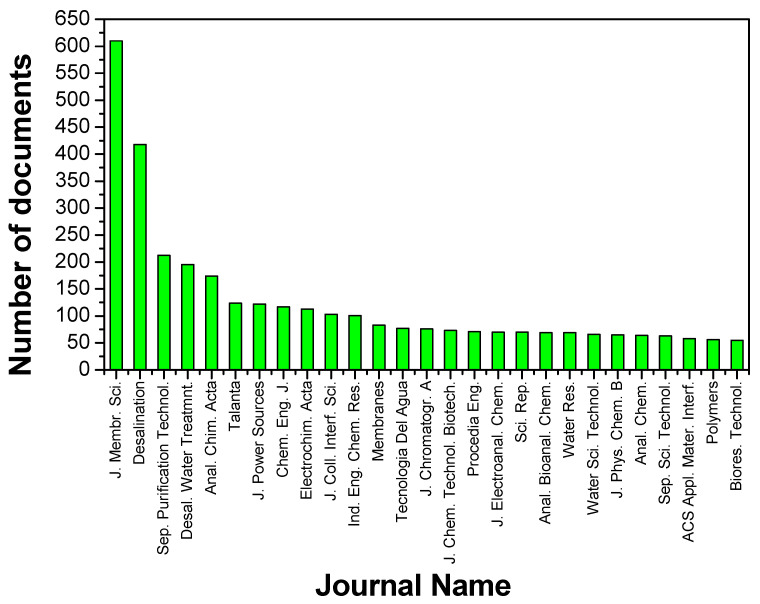
Spanish-based publications by the most productive early journals from 1960. (Source: Scopus, Keyword “Membrane” and Affiliation “Spain”).

The quality of the approach for this search can be observed by the leading journals and citations. In Figure 3, those 8707 papers have been classified by the name of the publication journal, and the 20 journals more represented are plotted in Figure 3.

## 2. Results and Discussion

### 2.1. Scientific Production of Spanish Researchers on Membrane Technology

As commented above, Figure 3 includes the most frequently encountered journals from 1960 to the moment of writing this review. Only those journals with more than 50 documents found in the sample analyzed in this work are represented in the figure. Most importantly, almost half of them are clearly recognized as reputed journal publications about membranes. In fact, *Journal of Membrane Science, Desalination* and *Separation and Purification Technology*, all Q1 journals and highly considered among the most reputed journals in the field, are the three most frequently encountered. It is worth noting that a very common journal during the early stages of Spanish membrane research, as *Journal of Non-equilibrium Thermodynamics*, is not represented in Figure 3 as in Figure 2, which shows that as years passed and more researchers started to work in membrane technology the focus of Spanish research moved from Irreversible Thermodynamics and charged membranes to the wide range of membrane processes appearing year by year, represented by chemical engineering and water treatment and separation technologies in general, or membrane materials and characterization in particular. 

**Figure 4:**
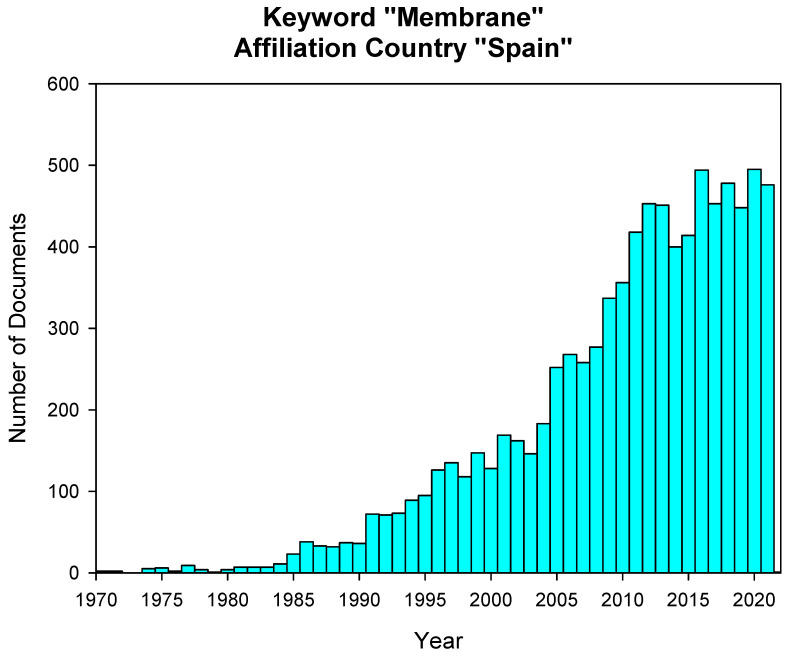
Spanish based publications in international journals from 1960. (Source: Scopus, Keyword “Membrane”).

On the temporal issue, Figure 4 presents the evolution in the total number of papers published per year during those last 50 years. A slow increase is observed in the first years (1975–1996), as was depicted above in Figure 1, but along the 90s the increase is continuous and uninterrupted (the number of papers rose from around 200 in 1995 to more than 600 in 2010). This increase corresponds to the already commented growth of Spanish research in general. It is obvious that a continuous increase in the funding devoted to research allowed more researchers to work in membrane science and technology. Moreover, more importantly, this allowed young researchers to have the opportunity to carry out significant parts of their work in leading research centers around the world. Thus, the obvious improvement in their training led to an increase in knowledge and expertise that they led back to their research groups after the return from these stays. Likewise, the increase in funds allowed greater participation of Spanish researchers in international congresses, favoring scientific exchange with top-level groups. The result is that a linear increase in funding translated into an almost exponential increase in scientific production, which also improved quality and visibility. Similarly, the figure also reflects the sudden slow-down of the pace of increasing the number of publications occurring during the economic crisis in 2010. The severe impact of the crisis and the subsequent cuts in research funding of the successive Spanish governments has not recovered and continues moving in almost a plateau shape (Figure 4).

A common indicator of the quality of a scientific article is the number of citations that the paper has received. Table 1 lists, in descending order, the 20 most cited articles from among those present in the non-exhaustive sample analyzed in this work. The papers included in this table are all very reputed works, which have attracted more than 400 citations each. Also noteworthy in this table is the fact that 12 out 20 of the most cited papers are reviews covering a wide range of membrane-related applications from traditional gas separation or water filtration to most recently, PEM fuel cells and other electrochemical applications using membranes. In the general sample analyzed, 82% of the documents retrieved are original scientific articles and only 6% were reviews. 

The 12 most productive journals (among those depicted in Figure 3) are further analyzed in Table 2, in decreasing order of the number of publications among JCR indexed journals. The most cited paper within the current search for each of these journals is also given in Table 2. The citations vary largely from one journal to another and largely depend on the date of publication and the age of the journal, encumbering fair comparison between the *Journal of Membrane Science* or *Desalination* with novel journals as *Membranes* or *Desalination and Water Treatment*, which were created in 2011. It reflects the scientific relevance of the journal, as most researchers try (as the first option) to publish their results in the top journals. Table 2, along with Figure 3, indicates that most of the research produced in membrane technology in Spain is published in the most recognized journals. However, it also reflects the historical background of the first established research groups dealing with membrane applications in industrial challenges such as wastewater treatment, as *Tecnología del agua* and *Water Research*, providing reviews highly consulted by their peers that have already been included in Table 1.

This significant amount of research publications in membrane technology in Spain covers almost all the fields of membranes, both fundamental and applied. Table 3 shows in fact that the major subject categories involved are Chemistry and Chemical Engineering, with transversal contributions from other categories as Materials and Environmental Science, which aim at the appointed potential of membrane technology as an alternative solution to existing processes in industry as will be discussed later. Furthermore, there is an important contribution of Biochemistry, Genetics and Molecular Biology that gives an idea of the close relation between membranes and Biological Sciences, although in this analysis, out of about 2700 documents, some could be surely more related with biological than synthetic membranes.

**Figure 5:**
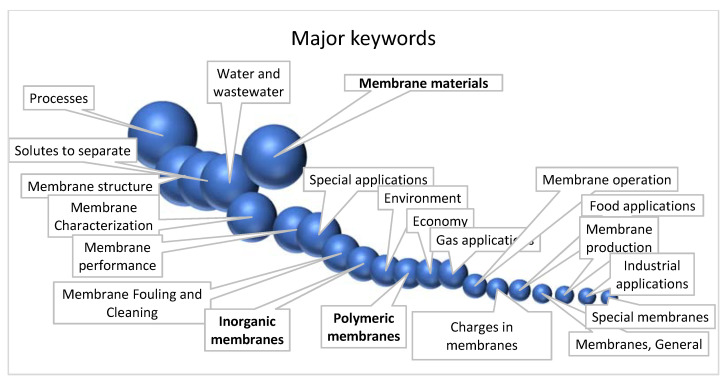
Comparative plot of relative number of papers according to the title keywords.

This can be complemented by a search on the feature words present in the title of the papers included in this database selection. We have counted the papers containing up to 200 keywords covering all aspects of membrane research. The entry “membrane materials” is highlighted because it involves inorganic and polymeric membranes, which are also represented separately. For the sake of simplicity, the results have been grouped in a shorter classification and they are presented, finally, in Figure 5.

Most of the papers selected referred in their title to one of the many membrane processes (1842, 21.1% of the total number of papers), being UF, RO, and NF the most frequently studied. Thus, 1412 papers (16.2%) are related to membrane structure and the different configurations a membrane can be made. There is also a significant number of papers devoted to fundamental research in membrane characterization (969, 11.1%) covering all analytical techniques available. The important contribution to the study of membrane fouling and cleaning (580, 6.7%), a key aspect of membrane applicability, cannot be missed from this review. There is also a significant number of papers devoted to membrane materials, which we have classified in this review as devoted to organic polymeric membranes (366, 4.2%) and inorganic or composite membranes (469, 5.4%). The fact that our selected sample identified more papers on inorganic than organic membranes, contrary to the practical reality, may be somehow surprising. This can be explained by the fact of the existence of some leading groups in ceramic membranes (in particular, the group from the University of Zaragoza, an international benchmark in zeolites and ceramics in general) that also attract a greater number of researchers and associated works. On the other hand, the classification of inorganic membranes contains also composite and mixed matrix membranes that have attracted growing interest in recent years.

Fundamental research can be completed with the 834 papers (9.6%) devoted to membrane performance, including studies on flux, rejection, permeability, or theoretical modelling of transport mechanisms.

Regarding the membrane applications, most of the studies dealt with several aspects of water and wastewater treatment (1338, 15.4%), including desalination or brackish water treatment. But many other membrane applications have been covered in these more than 40 years, such as: 185 papers on food applications, 119 on industrial applications, or 361 in gas applications. 

Last but not least, environmental studies are not forgotten (396 papers) covering all types of pollutants and contaminants. Finally, economic studies (365 papers), membrane production (135), charged membranes (197), or membrane operation (232) cover all aspects of membrane research. A great number of publications (1357, 15.6%) can be grouped under the term of different solutes to separate (remember effective separation is the key factor of membrane success).

**Figure 6:**
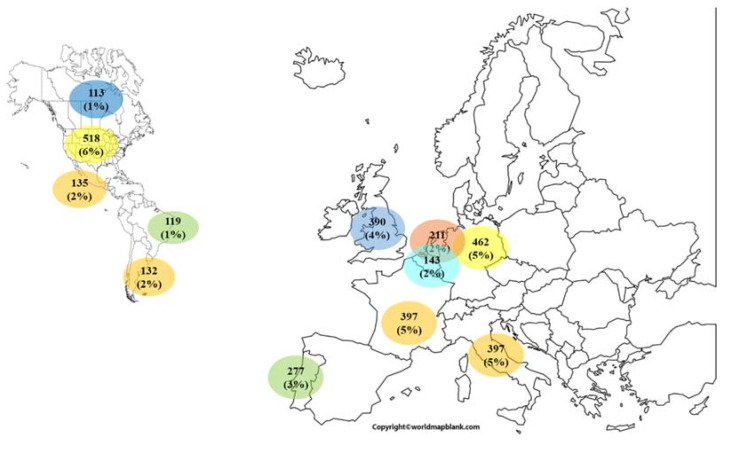
Top most collaborative countries with Spanish institutions worldwide.

Since research is a non-stand-alone process almost 60% of these works were performed in collaboration with researchers working at institutions in other countries, of which 6% were with the United States of America, 5% with Germany, France, or Italy, 4%, the United Kingdom and 3% Portugal. Figure 6 depicts the distribution of the most productive collaborations obtained from the present analysis search, above 2.4% of the total of the documents analyzed in this work. Collaborative works with Belgian institutions amount to 1.6% of the works delivered by the analyzed document samples analyzed in this work, same as the percentage of collaborations noted with Mexico or Argentina, which was to be expected in view of the historical and cultural connections between South American countries and Spain. There is in fact no general rule for international collaboration, but the common interest in a specific target and membrane application, as revealed by the polymer-inclusion membranes in environmental applications of the University of Girona with several institutions in France and Tunisia [60] and the University of Melbourne (Australia) [61]. To conclude with Figure 6, as expected, collaboration with European countries (taken together) is the most important (accounting for up to 60% of the total number of collaborative works) due surely to proximity reasons but, more importantly, to the impulse that European funding has given to international collaboration projects, through the various Framework Programs.

To conclude with this analysis of the Spanish publications on membranes and membrane processes, Table 4 shows the 10 most frequent authors in our list. For all of them, their affiliation is included, which represents the institution where the most productive research groups up to the moment of carrying out the present analysis belong. Several authors are currently affiliated with the same academic institution, which gives an idea of the size and continuity of the historical membrane science and technology research groups. It also reflects the fact that was mentioned earlier in this text, that Spanish research is mostly carried out in public universities and the Consejo Superior de Investigaciones Científicas (CSIC), the public council for scientific research. The creation of multiple mixed research institutes between the CSIC and a public university has contributed to the apparent preeminence of the CSIC in the membrane research field, in terms of the number of documents provided by the Scopus database in the current search (1207, 13.8%), which are shared with the universities also participating in the mixed research institutes, as the ITQ in Table 4. This collaborative combination allows concentrating and maximizing the research effort of the Universities.

Furthermore, the number of documents authored by them as reported in the present sample studied, as well as two parameters conventionally used to calibrate the scientific relevance of a research author. These parameters are the H-index and the total number of citations that this particular author has generated throughout his scientific production. The values of both parameters included in Table 4 are those provided by the Scopus search and are referred to the total number of publications of these authors in the database in the period considered in this work (1969–2021). Therefore, it should be kept in mind that, for some of these productive authors, research solely on membranes may only be a part of their total research on separation processes, while others with extensive publication on liquid membranes such as professor M. Valiente, from the Autonomous University of Barcelona, do not give more than 38 documents in the non-exhaustive search performed for this work, in contrast with the 189 documents on membranes a single search provides in Scopus. The same occurs with Dr. C. Fontás (from the University of Girona), presently the editor-in-chief of the section devoted to membrane analysis and characterization of the “Membranes” journal. This highlights the deficiency of the Scopus database for bibliometric analysis with a few selected keywords, being more focused on algorithms such as the H-index than the real research of the authors that must be evaluated manually and for 8707 documents delivered by the present study almost inapprehensible.

### 2.2. Outlook on Present and Future Development

In order to observe the continuity of the active authors and groups in the membrane science and technology field, the Supporting Information includes a directory of the research groups operational in membrane research and innovation. This directory has been formed thanks to the contribution of the interested actors and actresses themselves responding to a brief survey prepared on purpose for this work. The survey only asked for the institution, name, and acronym (when available) of the group, PhD-holding member components, and a brief description of their research lines on membranes and membrane processes. Some correspondents added some relevant results as the preferred papers published in the subject area, and these were completed on the writing of the manuscript including the most recent publications of each group for homogenization and update purposes.

From there, it is clear that as in other scientific fields, membrane research in Spain is still mainly conducted at national universities (19 institutions out of 24, see directory in the Supporting Information), specifically in departments of chemical engineering and applied chemistry. These departments have grown in the number of recipients over the years, mostly due to the lack of positions for trained researchers in other economic entities in Spain, and the atomization of membrane research into the specialty applications solving specific problems facing changing challenges of industry, health, and environment, as well as the advancements of materials characterization techniques. At least 15 groups report research lines or keywords devoted to technologies to improve environmental remediation or wastewater treatment using membranes and membrane processes. Of the groups from CSIC’s research institutes that responded to the survey, it should be considered the absence of ITQ (included in Table 4 above), and the usual collaboration with researchers from national universities, usually close in geographical proximity such as the University of Valladolid, along their historical development, thus strengthening the research efforts of both, as mentioned also in the previous section.

The size growth of the research groups on membrane science and development has led to the subdivision of the former departments into subgroups specialized in a certain approach of membrane research and the type of publications and journals their members divulgate their results on, belonging to a wide range of applications and membrane materials and module fabrication methods. See, for instance in the directory, the IEC and PROMETEO groups of the Polytechnic University of Valencia, distinguish themselves between electrochemical applications and membrane processes for water treatment [62,63,64], the subgroups of the GIQA group at Rey Juan Carlos University, one devoted to polymeric membranes for wastewater treatment, the other on inorganic membranes for gas separation [65,66,67], or the division of the Chemical and Biomolecular Engineering Department at the University of Cantabria into four subgroups covering almost all the membrane topics [68,69,70,71,72,73,74,75,76]. Likewise, the CREG group at the University of Zaragoza has been divided by the results obtained in the later years in two subgroups, one devoted to membrane reactors and inorganic membranes, and the other on the synthesis and characterization of mixed matrix membranes for molecular separations [77,78,79], while the consolidation of membranes in health issues such as bone regeneration, grows in groups similar to that from the University of Seville here [80,81,82]. 

The most commonly encountered are the membrane processes devoted to wastewater treatment, as Spain belongs to the Southern countries facing increasing scarcity of water supplies that will be aggravated by the actual climate emergency. In fact, desalination and water treatment industrial plants using membranes have already been implemented at a large scale for decades in Spain, with particular focus in the Mediterranean and Canary Islands. This follows the initial trend highlighted at the early period of the membrane technology field in international and Spanish research, where research was devoted to solving challenges faced in the industry and not as a technology in itself, which has not fully taken off in Spain yet.

## 3. Conclusions and Final Thought

The main challenge to membrane development is the persistent gap between high standard research performed at the scientific laboratories and the large-scale implementation, with no membrane company so far opened in the national territory This can be attributed to the general idiosyncrasy of Spanish economic, political, and historical development of the last century and the outcome of the economic crisis of 2010 whose effects we are still facing. Nevertheless, it is remarkable that most research groups active in membrane research—most of them present in the directory on the Supporting Information—are involved in European consortia or other internationally funded projects. The the recent announcement this year 2021 of a novel company producing metallic membranes for hydrogen purification, launched by the research technological center Tecnalia, may lead the way to a more active role of Spanish membrane research in the industrial development contribution to the national economic growth.

## Figures and Tables

**Table 1:** The articles cited more than 400 times.

Ranking	Most Cited Article	Type	Times Cited
1	Rodenas, T., Luz, I., Prieto, G., Seoane, B., Miro, H., Corma, A., Kapteijn, F., Llabrés i Xamena, F.X., Gascon, J. Metal-organic framework nanosheets in polymer composite materials for gas separation, Nature Materials, 14(1) 48–55, 2015	Article	1143
2	El-Bourawi, M.S., Ding, Z., Ma, R., Khayet, M. A framework for better understanding membrane distillation separation process, Journal of Membrane Science, 285, 4–29, 2006	Review	921
3	Navarro, R.M., Peña, M.A., Fierro, J.L.G. Hydrogen production reactions from carbon feedstocks: Fossil fuels and biomass, Chemical Reviews, 107(10) 3952–3991, 2007	Review	871
4	Moreira, F.C., Boaventura, R.A.R., Brillas, E., Vilar, V.J.P. Electrochemical advanced oxidation processes: A review on their application to synthetic and real wastewaters, Applied Catalysis B: Environmental, 202, 217–261, 2017	Review	868
5	Sunarso, J., Baumann, S., Serra, J.M., Meulenberg, W.A., Liu, S., Lin, Y.S., Diniz da Costa, J.C. Mixed ionic-electronic conducting (MIEC) ceramic-based membranes for oxygen separation, Journal of Membrane Science, 320, 13–41, 2008	Review	865
6	Mecerreyes, D. Polymeric ionic liquids: Broadening the properties and applications of polyelectrolytes, Progress in Polymer Science, 36(12) 1629–1648, 2011	Review	843
7	Garcia-Ochoa, F., Gomez, E. Bioreactor scale-up and oxygen transfer rate in microbial processes: An overview, Biotechnology Advances, 27(2) 153–176, 2009	Review	809
8	Radjenović, J., Petrović, M., Barceló, D. Fate and distribution of pharmaceuticals in wastewater and sewage sludge of the conventional activated sludge (CAS) and advanced membrane bioreactor (MBR) treatment, Water Research, 43(3) 831–841, 2009	Review	762
9	Khayet, M. Membranes and theoretical modeling of membrane distillation: A review, Advances in Colloid and Interface Science, 164, 56–88, 2011	Article	746
10	Heinzel, A., Barragán, V.M. Review of the state-of-the-art of the methanol crossover in direct methanol fuel cells, Journal of Power Sources, 84(1) 70–74, 1999	Review	742
11	Sirés, I., Brillas, E. Remediation of water pollution caused by pharmaceutical residues based on electrochemical separation and degradation technologies: A review, Environment International, 40(1) 212–229, 2012	Article	659
12	Klahr, B., Gimenez, S., Fabregat-Santiago, F., Hamann, T., Bisquert, J. Water oxidation at hematite photoelectrodes: The role of surface states, Journal of the American Chemical Society, 134(9) 4294–4302, 2012	Review	614
13	Ursúa, A., Gandía, L.M., Sanchis, P. Hydrogen production from water electrolysis: Current status and future trends, Proceedings of the IEEE, 100(2) 5898382, 410–426, 2012	Article	589
14	Petrović, M., Gonzalez, S., Barceló, D. Analysis and removal of emerging contaminants in wastewater and drinking water, TrAC - Trends in Analytical Chemistry, 22(10) 685–696, 2003	Conference Paper	523
15	Seoane, B., Coronas, J., Gascon, I., Benavides, M.E., Karvan, O., Caro, J., Kapteijn, F., Gascon, J. Metal-organic framework based mixed matrix membranes: A solution for highly efficient CO_2_ capture?, Chemical Society Reviews, 44(8) 2421–2454, 2015	Article	509
16	Asensio, J.A., Sánchez, E.M., Romero, P.-G. Proton-conducting membranes based on benzimidazole polymers for high-temperature PEM fuel cells. A chemical quest, Chemical Society Reviews, 39(8) 3210, 2010	Review	505
17	Pérez-González, A., Urtiaga, A.M., Ibáñez, R., Ortiz, I. State of the art and review on the treatment technologies of water reverse osmosis concentrates, Water Research, 46(2) 267–283, 2012	Review	489
18	Sorribas, S., Gorgojo, P., Téllez, C., Coronas, J., Livingston, A.G. High flux thin film nanocomposite membranes based on metal-organic frameworks for organic solvent nanofiltration, Journal of the American Chemical Society, 135(40) 15201–15208, 2013	Article	448
19	Falguera, V., Quintero, J.P., Jiménez, A., Muñoz, J.A., Ibarz, A. Title: Edible films and coatings: Structures, active functions and trends in their use, Trends in Food Science and Technology, 22(6) 292–303, 2011	Article	416
20	Quintana, J.B., Weiss, S., Reemtsma, T. Pathways and metabolites of microbial degradation of selected acidic pharmaceutical and their occurrence in municipal wastewater treated by a membrane bioreactor, Water Research, 39(12) 2654–2664, 2005	Review	411

**Table 2:** The top most productive journals in Figure 3.

Ranking	Journal	IF 2020 (WoS)	SJR 2020 (Scopus)	No. Documents (%)	Most Cited Article	Times Cited
1	Journal of Membrane Science	7.183	1.929	610 (7.0%)	El-Bourawi, M.S., Ding, Z., Ma, R., Khayet, M. A framework for better understanding membrane distillation separation process, J. Membr. Scie., 285, 4–29, 2006	921
2	Desalination	9.550	1.794	418 (4.8%)	Qtaishat, M., Matsuura, T., Kruczek, B., Khayet, M. Heat and mass transfer analysis in direct contact membrane distillation, Desal., 219, 272–292, 2008	318
3	Separation and Purification Technology	7.132	1.279	212 (2.4%)	Toledano, A., García, A., Mondragon, I., Labidi, J. Lignin separation and fractionation by ultrafiltration, Sep. Purif. Technol., 71(1) 38–43, 2010	318
4	Desalination and Water Treatment	0.854	0.251	195 (2.2%)	Albo, J., Luis, P., Irabien, A. Absorption of coal combustion flue gases in ionic liquids using different membrane contactors, Desal. Water Treatmnt., 27, 54–59, 2011	48
5	Analytica Chimica Acta	6.558	1.403	174 (2.0%)	Herrera-Herrera, A.V., González-Curbelo, M.T., Hernández-Borges, J., Rodríguez-Delgado, M.T. Carbon nanotubes applications in separation science: a review. Anal. Chim. A., 734, 1–30, 2012	205
6	Talanta	6.057	1.181	124 (1.4%)	Payán, M.R., López, M.A.B., Torres, R.F., Navarro, M.V., Mochón, M.C. Electromembrane extraction (EME) and HPLC determination of non-steroidal anti-inflammatory drugs (NSAIDs) in wastewater samples, Talanta, 85(1):394–399, 2011	103
7	Journal of Power Sources	8.247	2.139	122 (1.4%)	Heinzel, A., Barragán, V.M. Review of the state-of-the-art of the methanol crossover in direct methanol fuel cells, J. Power Sources, 84(1) 70–74, 1999	742
8	Chemical Engineering Journal	13.273	2.528	117 (1.3%)	Skouteris, G., Hermosilla, D., López, P., Negro, C., Blanco, Á. Anaerobic membrane bioreactors for wastewater treatment: A review, Chem. Eng. J., 198-199, 138–148, 2012	210
9	Electrochimica Acta	6.901	1.534	113 (1.3%)	Lobato, J., Cañizares, P., Rodrigo, M.A., Linares, J.J. PBI-based polymer electrolyte membranes fuel cells. Temperature effects on cell performance and catalyst stability, Electrochim. A., 52(12) 3910–3920, 2007	137
10	Journal of Colloid And Interface Science	8.128	1.538	103 (1.2%)	García-Payo, M.C., Izquierdo-Gil, M.A., Fernández-Pineda, C. Wetting study of hydrophobic membranes via liquid entry pressure measurements with aqueous alcohol solutions, J. Coll. Interf.ce Sci., 230(2) 420–431, 2000	163
11	Industrial and Engineering Chemistry Research	3.720	0.878	101 (1.2%)	Gadipelly, C., Pérez-González, A., Yadav, G.D., Ortiz, I., Ibáñez, R., Rathod, V.K., Marathe, K.V. Pharmaceutical industry wastewater: Review of the technologies for water treatment and reuse, Ind. Eng. Chem. Res., 53(29) 11571–11592, 2014	297
12	Membranes	4.106	0.609	83 (0.95%)	Blandin, G., Verliefde, A.R.D., Comas, J., Rodriguez-Roda, I., Le-Clech, P. Efficiently combining water reuse and desalination through forward osmosis-reverse osmosis (FO-RO) hybrids: A critical review, Membranes, 6(3) 10.3390/membranes6030037, 2016	61
13	Tecnología del Agua	-	0.209	77 (0.88%)	Artiga Acuña, P., Garcia-Toriello Romero, G., Garrido Fernández, J.M., Méndez Pampin, R. Membrane bioreactor: An advanced technology for the treatment and reuse of waste waters, Tecnologia del Agua, 26(269) 54–60, 2006	6
14	Journal of Chromatography A	4.390	1.011	76 (0.87%)	Rodil, R., Schrader, S., Moeder, M. Non-porous membrane-assisted liquid-liquid extraction of UV filter compounds from water samples, J. Chromatogr. A, 1216(24) 4887–4894, 2009	96
15	Journal of Chemical Technology and Biotechnology	2.750	0.650	73 (0.84%)	Rivera-Utrilla, J., Bautista-Toledo, I., Ferro-Garca, M.A., Moreno-Castilla, C. Activated carbon surface modifications by adsorption of bacteria and their effect on aqueous lead adsorption, J. Chem. Technol. Biotech., 76(12) 1209–1215, 2001	344
16	Procedia Engineering	1.880	0.320	71 (0.82%)	Stoller, M., Ochando-Pulido, J.M. Going from a critical flux concept to a threshold flux concept on membrane processes treating olive mill wastewater streams, Proc. Eng., 44, 607–608, 2012	41
17	Journal of Electroanalytical Chemistry	4.280	0.845	70 (0.80%)	Bisquert, J., Garcia-Belmonte, G., Fabregat-Santiago, F., Bueno, P.R. Theoretical models for ac impedance of finite diffusion layers exhibiting low frequency dispersion, J. Eletroanal. Chem., 475(2) 152–163, 1999	201
18	Scientific Reports	0.38 (*)	1.240	70 (0.80%)	Ivanova, M.E., Escolástico, S., Balaguer, M., Palisaitis, J., Sohn, Y.J., Meulenberg, W.A., Guillon, O., Mayer, J., Serra, J.M. Hydrogen separation through tailored dual phase membranes with nominal composition BaCe_0.8_Eu_0.2_O_3-δ_:Ce_0.8_Y_0.2_O_2-δ_ at intermediate temperatures, Sci. Rep., 6, 34773, 2016	32
19	Water Research	11.236	3.099	69 (0.79%)	Radjenović, J., Petrović, M., Barceló, D. Fate and distribution of pharmaceuticals in wastewater and sewage sludge of the conventional activated sludge (CAS) and advanced membrane bioreactor (MBR) treatment, Water Res., 43(3) 831–884, 2009	762
20	Analytical and Bioanalytical Chemistry	3.286	0.321	69 (0.79%)	Radjenovic, J., Petrovic, M., Barceló, D. Analysis of pharmaceuticals in wastewater and removal using a membrane bioreactor, Anal. Bioanal. Chem., 387(4) 1365–1377, 2007.	361

(*) 2-year impact factor.

**Table 3:** The top most popular subject categories.

Ranking	Subject Categories	Documents	Percentage (%) *
1	Chemistry	4366	50
2	Chemical Engineering	3425	39
3	Biochemistry, Genetics and Molecular Biology	2690	30
4	Materials Science	2455	28
5	Engineering	2088	24
6	Environmental Science	2043	23
7	Energy	799	9.2

* This percentage is calculated regarding the final 8707 records after scrutiny of the initial non-exhaustive search as explained in Section 1.2.

**Table 4:** List of 10 most prolific authors with their affiliation and relevance in the period 1960–2021 analyzed the present non-exhaustive overview performed in this work.

Ranking	Author	Current Affiliation	Documents	H-Index	Citations
1	Coronas, J.	Universidad de Zaragoza (UZ)	141	58	10,802
2	Ortiz, I.	Universidad de Cantabria (UC)	141	55	11,312
3	Khayet, M.	Universidad Complutense de Madrid (UCM)	135	60	11,320
4	Hernández, A.	Universidad de Valladolid (UVA)	115	37	4685
5	Benavente, J.	Universidad de Málaga (UMA)	92	28	2341
6	Prádanos, P.	Universidad de Valladolid (UVA)	92	35	3754
7	Urtiaga, A.	Universidad de Cantabria (UC)	90	43	5729
8	Irabien, A.	Universidad de Cantabria (UC)	88	52	9435
9	Téllez, C.	Universidad de Zaragoza (UZ)	88	48	7480
10	Serra, J.M.	Universidad Politécnica de Valencia, UPV—Consejo Superior de Investigaciones Científicas, CSIC (Instituto de Tecnología Química, ITQ)	77	39	5479

## Supplementary Materials

The following are available online at https://www.mdpi.com/article/10.3390/membranes11110808/s1.
