# Supplementary material for: Past, Present and Future of Membrane Technology in Spain
Source: Membranes (Basel). 2021 Oct 24;11(11):808. doi: 10.3390/membranes11110808 (PMC8625950; doi:10.3390/membranes11110808)
Supplement: Supplementary file 1 [file membranes-11-00808-s001.zip › membranes-1409007-SM.pdf]

membranes

Review

Past, present and future of membrane technology in Spain

José I. Calvo [1,*], Clara Casado-Coterillo [2,*] and Antonio Hernández [1]

1 Surfaces and Porous Materials (SMAP), Associated Research Unit to CSIC, UVainnova Bldg, P. Belén, 11 and Institute of Sustainable Processes (ISP), Dr. Mergelina, S/n, University of Valladolid, 47071, Valladolid, Spain
2 Department of Chemical and Biomolecular Engineering, Universidad de Cantabria, Av. Los Castros s/n, Santander 39005, Spain
* Correspondence: joseignacio.calvo@uva.es; clara.casado@unican.es

Supporting information. R+D+i Membrane Research Groups Spain

This Supporting information file contains the information of Spanish membrane research groups as received from them in a previous questionnaire. For all groups, the latest papers on membranes published (from Scopus) are included.

Basque Center for Macromolecular Design and Engineering -POLYMAT

Name (Acronym) of the group: NanoBioSeparations Group

Components: Thomas Schäfer

Location

Donostia/San Sebastián

Research lines

Description:

Stimuli-responsive membranes, mixed-matrix membranes, water treatment, membrane fouling detection, advanced surface-sensitive monitoring techniques, bioseparations, DNA-technology.

The NanoBioSeparations Group was founded with an ERC Starting Grant ("MATRIX") with the aim to develop a new concept of stimuli-responsive membranes and sensors based on functional DNA/membrane hybrid systems. It has an ample experience in advanced surface-sensitive techniques for characterizing molecular phenomena occurring at the membrane surface. Supported by an ERC Proof-of-Concept grant ("ESSENS") this has led to creating the startup "SURPHASE" (www.surphase.eu) which enables a unique early membrane fouling detection.

The Group co-organizes, together with João G. Crespo (Universidade NOVA de Lisboa), the successful bi-annual "Imagine Membrane" conference series in close collaboration between NAMS and EMS and is currently promoting the creation of an Iberian Membrane Working Group.

Apart from strict membrane-related activities, the NanoBioSeparations Group has been very active in discussing the future of young researchers in Europe with its PI serving as Chair of the COST Targeted Network "Sci-Generation" and as co-founder of the Young Academy of Europe (www.acadeuro.org).

Relevant results: [1-2]

Eurecat

Name (Acronym) of the group: UT Water, Air and Soil

Components: Sandra Casas, Edxon Licon, Gaetan Blandin, Federico Ferrari, Luca Sbardella, Carme Bosch, Xavier Martinez

Location

Cataluña

Research lines

Description:

Advanced processes for water and wastewater treatment, Desalination, Zero Liquid Discharge processes, Advanced separation processes in industrial production.

Relevant results: [3-5]

Institute of Polymer Science and Technology, ICTP-CSIC

Name (Acronym) of the group: Polycondensation and Polymer Membranes Group

Components: Cristina Álvarez and Ángel E Lozano

Location

Madrid

Research lines

Description:

Gas separation, olefin-paraffin purification, water purification, microporous polymers for CO_2 capture.

Gas separation, porous polymers, mixed matrix membranes, catalytic materials, polycondensation materials, heterocyclic materials, high thermal resistance membranes.

Relevant results: [6-8]

Polytechnic University of Catalunya

Name (Acronym) of the group: Resource Recovery Processes and Technologies (R2PT) as part of the Resource Recovery and Environmental Management (R2EM) research group

Components: Andriy Yaroshchuk , A.M. Sastre, Adriana Farrrán, Oriol Gibert, Cesar Valderrama, Mònica Reig, Julio López, Marc Fernández, José Luis Cortina

Location:

Multiscale Science and Engineering Research Center Escola de Enginyeria Barcelona Est (Barcelona TECH UPC), Barcelona

Research lines

R2PT group is developing research activity on development of sustainable urban and industrial waste management cycles based on resource recovery approaches promoting circular solutions of waste to product and waste to energy.

Relevant results: [9-11]

Politechnic University of Valencia

Name (Acronym) of the group: Grupo de Ingeniería Electroquímica y Corrosión, IEC

Components: Valentín Pérez-Herranz, Manuel César Martí-Calatayud, Emma Ortega-Navarro, Montserrat García-Gabaldón

Name (Acronym) of the group: Membrane Process and Environmental Effluent Treatment (PROMETEO)

Components: José Antonio Mendoza Roca, María Isabel Alcaina Miranda, Silvia Álvarez Blanco, José Miguel Arnal Arnal, Mª Amparo Bes Piá, Beatriz Elena Cuartas Uribe, Beatriz García Fayos, José Marcial Gozálvez Zafrilla, Alicia Iborra Clar, María Isabel Iborra Clar, Jaime Lora García, María José Luján Facundo, Francisco Juan Martínez Francisco, Antonio Diego Rodríguez López, María Pino Sancho Fernández, María Asunción Santafé Moros, María Cinta Vincent Vela

Location

Research Institute for Industrial, Radiophysical and Environmental Safety (ISIRYM), Valencia

Research lines

Research line IEC

Description:

Modification of membranes, electrodialysis, electromembrane processes, electrochemical reactors including membranes as separators, development of ceramic ion-exchange membranes.

Research line PROMETEO

2.1.Membrane processes applied to wastewater treatment. Membrane bioreactors.

2.2.Membrane processes applied to wastewater treatment. Recovery of high added value compounds. Circular economy

2.3.Wastewater reclamation

2.4. Synthetic membrane fabrication and modification

2.5. Manufacturing of low cost macroporous membranes

2.6. Simulation for the design and optimization of chemical, membrane and effluent treatment processes

2.7. Water treatment for developing countries

Relevant results: [12-14]

National Centre for Metallurgical Research. Spanish National Research Council (CSIC)

Name (Acronym) of the group: TecnoEco

Components: Francisco J. Alguacil; Félix A. López; Manuel Alonso; Esther Escudero

Location

Madrid

Research lines

Description:

Treatment of metal-bearing liquid solutions, metals including toxic and valuable elements. Metal recovery and recovery of critical metals

Relevant results:

64 publications in SCI Journals, such as [15-17]

Rey Juan Carlos University

Name (Acronym) of the group: Chemical and Environmental Engineering Group/ polymeric membranes (GIQA- PM)

Components: Arcadio Sotto Díaz; Jesús María Arsuaga Ferreras

Name of the group: Chemical and Environmental Engineering Group / inorganic membranes (GIQA- IM)

Components: D. Alique, J.A. Calles, R. Sanz, D. Martínez-Díaz, D. Sanz, K. Cruz

Location

Madrid

Research lines

Research line GIQA/PM

Description: Synthesis and characterization of nanocomposite membranes; Functional and physico-chemical membrane characterization; Ultrafiltration, Nanofiltration and Reverse Osmosis; Diafiltration

Research line GIQA/ IM

Description: Development of Pd-based membranes onto tubular Porous Stainless-Steel Supports for separation of ultra-pure hydrogen. Modifications of commercial porous substrates and synthesis of the H_2-selective Pd-film by Electroless Pore-Plating to increase the mechanical resistance of the composite membrane while high perm-selectivity to hydrogen is maintained. Pd-films thinner than 10 micrometers with strong adherence under operating conditions on both external and internal side of tubular supports. Process intensification by combining membranes with catalysts in a same unit to perform reforming and WGS reactions.

Relevant results: [18-20]

TECNALIA

Name (Acronym) of the group: Membrane Technology & Process Intensification

Components: https://www.tecnalia.com/tecnologias/tecnologia-de-membranas-e-intensificacion-de-procesos

Location

San Sebastián (Guipuzcoa)

Research lines

Description: At Tecnalia Membrane Technology and Process Intensification department, we develop advanced membranes and membrane reactors for Industrial Gas separation demands. Aiming at increasing the efficiency, reducing the operating and capital costs as well as physical and environmental footprint in different applications. Some examples of research fields are: Hydrogen purification and production, CO_2 capture and conversion, natural gas processing, biogas upgrading and olefin/paraffin separation among others. We have extensive testing and membrane manufacturing capabilities and we have experience on working from proof-of-concept to relevant industrial scale.

At the Membrane Technology & Process Intensification department of Tecnalia we are mainly focused on the development of both polymeric as well as inorganic membranes for the different applications in order to bridge the gap from lab to industry. In addition, we have an strategic agreement with the University of Eindhoven, expert in process design and simulation together with expertise in the field of Membrane reactors.

TUe and Tecnalia form a unique team that takes advantage of synergistical effect between membranes and reactors.

Together, we share a team of more than 25 researchers that cover the entire value chain around process intensification through membrane reactors: Process design & optimization, membrane and reactor development and scale up, prototype development and validation and Techno-economic analysis.

Relevant results:

The team gathers more than 10 years collaborating together in the frame of 8 European projects as well as uncountable private initiatives. We own several patents, more than 50 common publications in peer reviewed journals as well as several book chapters. As result of our collaboration, several patents have been submitted and a membrane-based company has been created called H2SITE [21-23].

Universidad Autónoma de Madrid

Name (Acronym) of the group: Electrochemistry Research Group /GIE2Q, Department of Applied Physics

Components: Pilar Ocón Esteban, Juan Ramón Avilés Moreno

Location

Madrid

Research lines

Description:

- Study of corrosion in alloys and metallic materials for industrial use.
- Development of electrocatalytic materials for use in polymeric membrane fuel cells. PEMFC
- Synthesis and characterization of ion exchange membranes for battery applications.
- Evaluation and development of advanced Pb/acid batteries.
- Study of Metal / Air batteries.

Relevant results: [24-26]

University of Cádiz

Name (Acronym) of the group: Applied Separation Techniques

Components: José A. López López, Carolina Mendiguchía Martínez, Carlos Moreno Aguilar (PI), Juan J. Pinto Ganfornina, Araceli Rodríguez Romero

Location

Cádiz

Research lines

Description:

Liquid Membranes and hollow fiber liquid phase microextraction. Developments of new systems and application to sample preparation.

Relevant results: [27-29]

Universidad de Cantabria

1. *Name (Acronym) of the group:* Advanced Separation Processes (PAS)

   Components: Inmaculada Ortiz, Daniel Gorri, Eugenio Bringas, Alfredo Ortiz, Marcos Fallanza, Lucía Gómez, Pedro Gómez. http://grupos.unican.es/pasep

2. *Name (Acronym) of the group:* Environmental technologies and Bioprocesses (ETB)

   Components: Ane Urtiaga, Nazely Diban, Gabriel Zarca, Fernando Pardo. https://grupos.unican.es/tab/

3. *Name (Acronym) of the group:* Development of Chemical Processes and Pollutant Control (DEPRO-Membrane Processes)

   Components: Aurora Garea, Clara Casado Coterillo, Angel Irabien, https://grupos.unican.es/depro/

4. *Name (Acronym) of the group:* Sustainable Process Engineering group (SPE)

   Components: Raquel Ibáñez Mendizabal, Maria Fresnedo San Román, Marta Herrero Gonzalez, Gema Pérez.

Location

Department of Chemical and Biomolecular Engineering, Santander (Cantabria)

Research lines

1. Research line PAS

Description:

1. High performance materials for microfluidic and micro-magnetoforetic separations

1.1. Facilitated transport membranes for olefin/paraffin separation

1.2. Hydrogen economy: high performance membranes for PEMFC

1.3. Selective functionalized membranes with hollow fiber configuration for fluid phase molecular separations

1.4. Reverse electrodialysis for the recovery of saline gradient energy

1.5. Pervaporation. Biofuels.

2. Research line ETB

Description:

2.1 Gas Separation. Hydrogen recovery and synthesis gas upcycling. Separation of refrigerants.

2.3 Reverse osmosis, nanofiltration and ultrafiltration. Water treatment.

2.4 Membrane contactors. Supported liquid membranes. Materials recovery.

2.5 Biocompatible membranes for tissue engineering, drug delivery and biomedical applications. Perfusion bioreactors.

2.6 Membrane fabrication: Electrospinning, solvent induced phase separation (SIPS), casting of thin film composite ionic liquid membranes.

2.7 Mathematical modelling. Thermodynamic modelling of SIPS. Process integration.

3. Research line DEPRO

Description:

Carbon Capture and Utilisation. Circular Economy Electrochemical Technologies. Membrane Processes

3.1. Research and Development of Membrane Separation Processes

3.2. Sustainable Process Intensification with Membranes

4. Research line SPE

4.1.Development of membrane technologies for food industry and water reuse

4.2.Modeling, simulation and optimization of separation processes with liquid membranes

4.3.Development of electromembrane technologies for industry and environmental applications

Research line DEPRO

Carbon Capture and Utilisation. Circular Economy Electrochemical Technologies. Membrane Processes

3.1. Research and Development of Membrane Separation Processes

3.2. Sustainable Process Intensification with Membranes

Research line SPE

4.1.Development of membrane technologies for food industry and water reuse

4.2.Modeling, simulation and optimization of separation processes with liquid membranes

4.3.Development of electromembrane technologies for industry and environmental applications

Relevant results: [30-38]

University Complutense of Madrid

Name (Acronym) of the group: Membranes and Renewable Energies (MER)

Components: Mohamed Khayet; Carmen García Payo; Loreto García Fernández; Julio Antonio Sanmartino; Esther García Merino; Jorge Contreras Martínez; Alia Baroudi; Miguel Ángel López Manchado

Location

Madrid

Research lines

Description: The actual interests of the MER research group in the field of membrane science and nanotechnology is desalination, water treatment, nanostructured membranes for different membrane separation processes (Membrane Distillation, Forward Osmosis, Micro- Ultra- and Nano-filtration, Reverse Osmosis, Electrodialysis and its variants, Capacitive Deionization, Pervaporation, etc.), design and preparation of different types of membranes (phase inversion flat sheet, hollow fiber, nanofiber, 2D-membranes, photothermally heated membranes, green membranes, etc.), Design and preparation of membrane modules, nanocomposites and nanofluids, theoretical modelling, optimization of membrane separation plants, solar-driven separation processes, etc.

Relevant results:

For more information visit the following links:

https://www.ucm.es/membrane-energy-group/

https://scholar.google.com/citations?user=H_lnjFkAAAAJ&hl=en

Such as: [39-41]

University of Girona

1. *Name (Acronym) of the group:* Lequia (Laboratory of Chemical and Environmental Engineering)

   Components*:* Ignasi Rodriguez-Roda, Jesus Colprim, Joaquim Comas, Manel Poch, Maria Dolors Balaguer, Maria Martin, Marta Vedaguer, Sebastia Puig, Alexandra Popartan, David Palma, Alba Cabrera, Albert Magrí, Hector Monclus, Helena Matabosh, Narcis Pous, Pau Batlle, Raquel Garcia-Pacheco, Lluis Godo, Silvia Bolognesi

2. *Name (Acronym) of the group:* Química Analítica y Ambiental (QAA)

   Components*:* Clàudia Fontàs, Victòria Salvadó, Enriqueta Anticó, Manuela Hidalgo

Location

Girona

Research lines

Description:

1. Membrane bioreactors; End-of-life membrane reuse; Forwards Osmosis; Decentralised system for water purification; Control system and Artificial Intelligence for membrane processes optimization; Decision support systems; Water Reuse; Water Desalination; drinking water.

2. Separation processes based on functionalized membranes; supported liquid membranes; polymer inclusion membranes; membranes for metals' speciation and monitoring in natural waters; membrane systems for the clean-up of natural waters and industrial wastewaters; physico-chemical and electrical characterization of membranes; environmental and industrial sampling and analysis.

Relevant results: [42-47]

University of Granada

1.- Name (Acronym) of the group: Bioreactores (BIO110)

Components: Emilia M. Guadix; Antonio Guadix, M. Carmen Almecija, Raúl Pérez-Gálvez , F. Javier Espejo-Carpio, Pedro J. García-Moreno

2.- Name (Acronym) of the group: TEP025

Components: Antonio Martínez-Ferez; Javier M. Ochando-Pulido

Location

Granada

Research lines

1.- Description: High performance tangential flow filtration (Concentration and purification of biomolecules by ultra and nanofiltration)

2.- Description: wastewater purification, isolation of bioactive compounds.

Relevant results: [48-53]

University of Las Palmas de Gran Canaria

Name (Acronym) of the group: SEIP

Components: Ignacio de la Nuez Pestana, Alejandro Ruiz García, José Jaime Sadhwani Alonso, Juan José Santana Rodriguez

Location

Las Palmas

Research lines

Description: Desalination, Modeling, Fouling

Relevant results: [54-56]

University of Málaga

Name (Acronym) of the group: Comportamiento y procesado de materiales

Components: Antonio González Herrera, José Manuel García-Manrique Ocaña, Antonia María Lima Rodríguez

Location

Málaga

Research lines

Description: We have been studying the acoustic-mechanics response of the hearing system for several years. A key element in this system is the tympanic membrane.

Uncertainties and difficulties to properly modelling its behaviour led us to develop specific research on the basic properties of the membrane. Mainly in the field of sound transmission. Both numerical and experimental studies have been developed.

One of the main goals of this research line is the characterization of the mechanical properties of the membrane.

Relevant results: [57-59]

University of Murcia

Name (Acronym) of the group: Análisis y Simulación de Procesos Químicos, Bioquímicos y de Membrana

Components: Elisa Gómez Gómez, Josefa Bastida Rodríguez, María Fuensanta Máximo Martín, Asunción María Hidalgo Montesinos, María Claudia Montiel Morte, María Gómez Gómez, María Dolores Murcia Almagro, Salvadora Ortega Requena, Mar Serrano Arnaldos, Clara Yagüe Gómez, José Luis Gómez Carrasco, Antonio Bódalo Santoyo, Dalje Sunith Barbosa Trillos, Daniel Delgado Torrico, Arturo Sánchez Martínez, José Antonio Macario Legaz

Location

Murcia

Research lines

Description:

1. Membrane processes for desalination, regeneration and treatment of liquid effluents.

2. Separation of bioactive compounds by membrane processes

Relevant results: [60-62]

University of Oviedo

Name (Acronym) of the group: MPH

Components: José R. Álvarez, Susana Luque, Gemma Gutiérrez, María Matos, Sonia Álvarez

Location

Asturias

Research lines

Description: Our research is primarily focused on the application of the membrane technology and hybrid processes that derive from it, to industrial and environmental problems. With this objective, we carry out from basic studies (for a better knowledge of the fundamentals of the techniques) to pilot tests (in industrial plants). The data obtained from the experimentation in pilot scale systems allow us better designs and more realistic economic evaluations for the scale-up.

Relevant results: [62-65]

Universidad del País Vasco

Name (Acronym) of the group: Labquimac

Components: Jose Luís Vilas-Vilela, Leire Ruiz Rubio, Jose Manuel Laza, Leyre Pérez, Isabel Moreno, Antonio Veloso

Location

Bilbao (Vizcaya)

Research lines

Description:

Materials for environmental remediation

Relevant results: [65-68]

Universidad Politécnica de Cartagena (UPCT)

Name (Acronym) of the group: Química y Tecnología-Membranas/ QUIMYTEC-MEMBRANAS

Components: Beatriz Miguel Hernández; Gerardo León Albert

Location

Cartagena

Research lines

Description:

1. Pollutants removal by liquid membranes, polymer inclusion membranes and driven membrane processes (reverse osmosis, nanofiltration and ultrafiltration).

2. Membranes modification by graphene-based nanomaterials.

Relevant Results:

1. Kinetics models in bulk liquid membrane processes

2. Transport studies in emulsion liquid membranes

3. Novel supported liquid membranes prepared by ultrasound

4. Modelling transport process in reverse osmosis and nanofiltration

5. Membrane modification (reverse osmosis, nanofiltration, ultrafiltration and polymer inclusion membranes) by graphene-based nanomaterials.

[69-71]

Universitat Rovira i Virgili (URV)

Name (Acronym) of the group: Food, Innovation & Engineering (FoodIE)

Components: Montserrat Ferrando, Sílvia de Lamo-Castelleví, Carme Güell

Location

Tarragona

Research lines

Description:

1. Membrane fouling characterization by CSLM and ATR-IRMS during filtration processes of biological streams.

2. Low temperature concentration by forward osmosis of aqueous solutions.

3. Membrane/microporous emulsification processes for encapsulation of food ingredients

Relevant Results:

1. Mechanisms of protein fouling during microfiltration with polymeric membranes.

2. Low energy strategies for valorization of food byproducts

3. Encapsulation of aromas and bioactive ingredients using by single, multiple emulsions, and emulsion-based microcapsules.

4. Low energy microporous emulsification: influence of conventional and new emulsifiers in emulsion stability and productivity of the system.

5. Influence of operation parameters in membrane emulsification performance.

[72-74]

Universidad de Valladolid

Name (Acronym) of the group: SMAP

Components: Antonio Hernández, Pedro Prádanos, José I. Calvo, Laura Palacio, Ángel Lozano, Javier Carmona, Bibiana Comesaña, Cenit Soto, Mónica Salamanca

Location

Facultad de Ciencias, Valladolid (Spain)

www.smap.uva.es

Research lines

Description:

1.1 New materials for Gas separation and CO_2 absorption.

1.2 Modifications of structure and surface properties of membranes to increase permeability or selectivity and to decrease fouling, plasticization or ageing.

1.3 Electrical properties of membrane surfaces through: Impedance spectroscopy, true and adsorbed charges, zeta potentials etc...

1.4 Structural characterization by: SEM and TEM microscopy and computerized image analysis and AFM microscopy.

1.5 Hg intrusion porosimetry; Air permeation porometry; Gas adsorption-desorption and other porometric techniques.

1.5 Fouling kinetics and corresponding dynamical changes of structure.

Other related areas where the group is or has been active include:

1.6 Air and water quality and treatment of industrial and urbane effluents,

1.7 Food applications of micro, ultra and nanofiltration,

Relevant results: [75-77]

University of Seville

Name (Acronym) of the group: CTS-523 - Innovacion y Desarrollo en Tecnicas y Fundamentos de Cirugia Bucal y Craneofacial

Components: Daniel Torres-Lagares, José Luis Gutiérrez Pérez, David Gallego Romero, José María Hernández Guisado, Angela Rodríguez-Caballero, Juan David Gonzalez Padilla, María Rizo Gorrita, M Isabel González Martín, Pilar Hita Iglesias

Location

Sevilla

Research lines

Description: Advanced polymeric membranes and their role in bone regeneration

Relevant results: [78-80]

Universidad de Zaragoza

Name (Acronym) of the group: Catalysis, molecular separations and reactor engineering group (CREG-I3A)

Components: Miguel Menéndez, Javier Herguido, Jose Angel Peña, Jaime Soler

Name (Acronym) of the group: Membranes and Nanostructured Materials (CREG-INMA)

Components: Joaquín Coronas; Carlos Téllez; Óscar de la Iglesia; Patricia Gorgojo; Beatriz Zornoza; Magdalena Malankowska; Lorena Paseta

Location

Zaragoza

Research lines

Research line CREG-I3A

Description: Membrane reactors (for catalytic oxidation, methanol or Fischer-Tropsch synthesis, esterifications), zeolite membranes, Pd membranes, MOF membranes.

Research line CREG-INMA

Description: Synthesis and characterization of nanoporous materials: zeolites, titanosilicates, layered materials, ordered mesoporous silicas, etc., and more recently graphene derivatives, MOFs and COFs controlling its surface chemistry and textural properties.

- Development of mixed matrix membranes: dense and supported on flat and hollow fiber polymeric supports for molecular separations.

- Application of nanostructured materials to membranes to enhance their separation ability: gas separation (CO_2 capture, H_2 purification, etc.), nanofiltration (solvent purification, removal of micropollutants from water), pervaporation, membrane distillation and membrane reactors.

Relevant results:

Publications available at https://i3a.unizar.es/es) and https://inma.unizar-csic.es/, such as [80-83]

References

1. Rivilla, I., Aparicio, B., Bueno, J.M., Casanova, D., Tonnelé, C., Freixa, Z., Herrero, P., Rogero, C., Miranda, J.I., Martínez-Ojeda, R.M., Monrabal, F., Olave, B., Schäfer, T., Artal, P., Nygren, D., Cossío, F.P., Gómez-Cadenas, J.J.; Fluorescent bicolor sensor for low-background neutrinoless double beta decay experiments; Nature, 583 (2020) 48.
2. Schäfer, T., Özalp, V.C..; DNA-Aptamer Gating Membranes; Chemical Communications, 51 (2015) 1285
3. Monneron--Gyurits M., Soubrand M., Joussein E., Courtin- A. Nomade, Jubany I., Casas S., Bahi N., Faz A., Gabarron M., Acosta J.A., Martinez-Martinez S., Investigating the relationship between speciation and oral/lung bioaccessibility of a highly contaminated tailing: contribution in health risk assessment, Environmental Science and Pollution Research, 27 (2020) 40732-40748.
4. Blondet I., Schreck E.,Viers J., Casas S., Jubany I., Bahi N., Zouiten C., Dufrechou G., Freydier R., Galy-Lacaux C., Martinez-Martinez S., Faz A.; Soriano-Disla M., Acosta J.A., Darrozes J.; Atmospheric dust characterisation in the mining district of Cartagena-La Unión, Spain: Air quality and health risks assessment; Science of the Total Environment 693 (2019) 133496.
5. Gibert O., Vera M.,Cruz S., Boleda M.R., Paraira M., Martin-Alonso J., Casas S., Bernat X.; Characterisation of organic foulants on full-scale UF membranes during filtration, backwash and chemical cleaning episodes; Desalination and Water Treatment 89 (2017) 17-28
6. Esteban N., Ferrer M.L., Ania C.O., De La Campa J.G., Lozano A.E., Alvarez C., Miguel J.A.; Porous Organic Polymers Containing Active Metal Centers for Suzuki-Miyaura Heterocoupling Reactions; ACS Applied Materials and Interfaces, 12 (2020) 56974-56986.
7. Alvarez C., Lozano A.E., Juan-y-Seva M., de la Campa J.G.; Gas separation properties of aromatic polyimides with bulky groups. Comparison of experimental and simulated results; Journal of Membrane Science, 602 (2020) 117959.

8. Soto C., Aguilar Lugo C., Rodriguez S., Palacio L., Lozano A.E., Pradanos P., Hernandez A.; Enhancement of CO_2/CH_4 permselectivity via thermal rearrangement of mixed matrix membranes made from an o-hydroxy polyamide with an optimal load of a porous polymer network; Separation and Purification Technology 247 (2020) 116895.
9. Reig, M; Vecino, X; Cortina, J L. Arsenic impact on the valorisation schemes of acidic mine waters of the Iberian Pyrite Belt: Integration of selective precipitation and spiral wound nanofiltration processes. Journal of hazardous materials, 2021, 403, 123886. 05/02/2021.
10. D'Haese, A.; Licon, E.; Yaroshchuk, A.; Model of forward osmosis through composite/asymmetric membranes: effects of support inhomogeneity and solution non-ideality, Journal of Membrane Science, 602 (2020).
11. Pavon, S.; Fortuny, A.; Coll, M.T.; Becue-Bertaut, M.; Sastre, A., Permeability dependencies on the carrier concentration and membrane viscosity for Y(III) and Eu(III) transport by using liquid membranes, Separation and Purification Technology,239 (2020) 16573.
12. Rotta E.H., Marder L., Perez-Herranz V., Bernardes A.M.; Characterization of an anion-exchange membrane subjected to phosphate and sulfate separation by electrodialysis at overlimiting current density condition; Journal of Membrane Science, 635 (2021) 119510.
13. Cifuentes-Cabezas M., Carbonell-Alcaina C., Vincent-Vela M.C., Mendoza-Roca J.A., Alvarez-Blanco S.; Comparison of different ultrafiltration membranes as first step for the recovery of phenolic compounds from olive-oil washing wastewater; Process Safety and Environmental Protection, 149 (2021) 724-734.
14. Catalán-Martínez, D., Santafé-Moros, A., Gozálvez-Zafrilla, J.M., García-Fayos, J., Serra, J.M., Characterization of oxygen transport phenomena on BSCF membranes assisted by fluid dynamic simulations including surface exchange, Chemical Engineering Journal,387 (2020) 124069, 1-15.
15. Robla J., Garcia-Hierro J., Alguacil F.J., Dittami S.M., Marie D., Villa E., Deragon E., Guillebault D., Mengs G., Medlin L.K.; Determination of the efficiency of filtration of cultures from microalgae and bacteria using hollow fiber filters; Environmental Science: Water Research and Technology, 7 (2021) 1230-1239.
16. Alguacil F.J., Alcaraz L., Largo O.R., Lopez F.A.; Transport of Au(III) from HCl medium across a liquid membrane using $R_3NH^+ Cl^-$ /toluene immobilized on a microporous hydrophobic support: Optimization and modelling; Membranes, 10 (2020) 1 – 13.
17. Alguacil F.J., Lopez F.A.; Dispersion-free extraction of In(III) from HCl solutions using a supported liquid membrane containing the HA324H+Cl− ionic liquid as the carrier; Scientific Reports, 10 (2020) 13868.
18. Qiu Y., Lv Y., Tang C., Liao J., Ruan H., Sotto A.; Sustainable recovery of high-saline papermaking wastewater: Optimized separation for salts and organics via membrane-hybrid process, Shen J. Desalination, 507 (2021) 114938.
19. Diez B., Sotto A., Martin A., Arsuaga J., Rosal R.; Poly(vinyl chloride)-hyperbranched polyamidoamine ultrafiltration membranes with antifouling and antibiofouling properties; Reactive and Functional Polymers, 154 (2020) 104669.
20. Martinez-Diaz, Leo, P., Sanz, R., Carrero, A,, Calles, J.A., Alique, D. Life cycle assessment of H_2-selective Pd membranes fabricated by electroless pore-plating, Journal of Cleaner Production, 316 (2021) 128229.
21. Cechetto V., Di Felice L., Medrano J.A., Makhloufi C., Zuniga J., Gallucci F.; H2 production via ammonia decomposition in a catalytic membrane reactor: Fuel Processing Technology, 216 (2021) 106772.
22. Fernandez E., Helmi A., Medrano J.A., Coenen K., Arratibel A., Melendez J., de Nooijer N.C.A., Spallina V., Viviente J.L., Zuniga J., van Sint Annaland M., Pacheco Tanaka D.A., Gallucci F.; Palladium based membranes and membrane reactors for hydrogen production and purification: An overview of research activities at Tecnalia and TU/e; International Journal of Hydrogen Energy, 42 (2017) 13763-13776.
23. Fernandez-Barquin A., Casado-Coterillo C., Etxeberria-Benavides M., Zuniga J., Irabien A.; Comparison of Flat and Hollow-Fiber Mixed-Matrix Composite Membranes for CO_2 Separation with Temperature; Chemical Engineering and Technology, 40 (2017) 997-1007
24. Charradi, K., Ahmed, Z., Thmaini, N., Aranda, P., Al-Ghamdi, Y.O., Ocon, P., Keshk, S.M.A.S., Chtourou, R. Incorporating of layered double hydroxide /sepiolite to improve the performance of sulfonated poly(ether ether ketone) composite membranes for proton exchange membrane fuel cells, Journal of Applied Polymer Science, 138(9) (2021) 50364.
25. Herranz, D., Coppola, R.E., Escudero-Cid, R., Ochoa-Romero, K., D'Accorso, N.B., Pérez-Flores, J.C., Canales-Vázquez, J., Palacio, C., Abuin, G.C., Ocón, P. Application of crosslinked polybenzimidazole-poly(Vinyl benzyl chloride) anion exchange membranes in direct ethanol fuel cells, Membranes, 10(11) (2020) 349, pp. 1-19
26. Coppola, R.E., Herranz, D., Escudero-Cid, R., Ming, N.B., D'Accorso, N.B., Ocón, P., Abuin, G.C. Polybenzimidazole-crosslinked-poly(vinyl benzyl chloride) as anion exchange membrane for alkaline electrolyzers, Renewable Energy, 157 (2010) 71-82.
27. Gonzalez-Alvarez R.J., Pinto J.J., Lopez-Lopez J.A., Mendiguchia C., Moreno C.; Selective solvent bar micro-extraction as a single-step approach for the measurement of Cu fractions in seawater; Analytical and Bioanalytical Chemistry, 412 (2020) 1863-1870
28. Ahechti M., Benomar M., El Alami M., Mendiguchia C.; Metal adsorption by microplastics in aquatic environments under controlled conditions: exposure time, pH and salinity; International Journal of Environmental Analytical Chemistry (2020) doi: 10.1080/03067319.2020.1733546.
29. Silva M., Mendiguchia C., Moreno C.; Key factors in electromembrane microextraction systems for metals analysis in natural waters; International Journal of Environmental Analytical Chemistry, 98 (2018) 1388-1397

30. Basauri, A., González-Fernández, C., Fallana, M., Bringas, E., Fernández-López, ¨R., Giner, L., Moncalián, G., Cruz, F. de la, Ortiz, I. Biochemical interactions between LPS and LPS-binding molecules, Critical Reviews in Biotechnology, 40(3) (2020) 292-305.
31. Yañez M., Ortiz A., Gorri D., Ortiz I.; Comparative performance of commercial polymeric membranes in the recovery of industrial hydrogen waste gas streams; International Journal of Hydrogen Energy, 46 (2021) 17507-17521.
32. Corredor, J., Perez-Peña, E., Rivero, M.J., Ortiz performance of rgo-tio2 photocatalytiuc membranes for H_2 separation, Membranes, 10(9) 218 (2020) 1-13.
33. Pardo F., Gutierrez-Hernandez S.V., Zarca G., Urtiaga A.; Toward the Recycling of Low-GWP Hydrofluorocarbon/Hydrofluoroolefin Refrigerant Mixtures Using Composite Ionic Liquid-Polymer Membranes; ACS Sustainable Chemistry and Engineering, 9 (2021) 7012-7021.
34. Arguillarena, A., Margallo, M., ARrruti-Fernandez, A., Pinedo, J., Gomez, P., Urtiaga, A. Scale-up of membrane-based zinc recovery from spent pickling acids of hot-dip galnizing. Membranes, 10(12) 444 (2020).
35. Abejón, R., Fernández-Ríos, A., Domínguez-Ramos, A., Laso, J., Ruiz-Salmón, I., Yáñez, M., Alfredo, A., Gorri, D., Donzel, N., Jones, D., Irabien, A., Ortiz, I., Aldaco, R., Margallo, M. Hydrogen recovery from waste gas streams to feed (High-temperature PEM) fuel cells: Environmental performance under a Life Cycle thinking approach. Applied Sciences, 10(21) 4761 (2020).
36. Vadillo, J.M., Hospital-Benito, D., Moya, C.; Gomez-Coma, L., Palomar, J., Garea, A., Irabien, A. Modelling and simulation of hollow fiber membrane vacuum regeneration for CO_2 desorption process using ionic liquids, 277 (2021) 119465.
37. García-Cruz, L., Casado-Coterillo, C., Iniesta, J., Montiel, V., Irabien, A. Chitosan:poly(vinyl)alcohol composite alkaline membrane incorporating organic ionomers and layered silicate materials into a PEM electrochemical reactor, Journal of Membrane Science, 498 (2016) 395-407.
38. Fernandez-Gonzalez C., Zhang B.b, Dominguez-Ramos A, Ibanez R., Irabien A., Chen Y. Enhancing fouling resistance of polyethylene anion exchange membranes using carbon nanotubes and iron oxide nanoparticles. Desalination, 411 (2017) 19-27.
39. Essalhi M., Khayet M., Tesfalidet S., Alsultan M., Tavajohi N.; Desalination by direct contact membrane distillation using mixed matrix electrospun nanofibrous membranes with carbon-based nanofillers: A strategic improvement; Chemical Engineering Journal, 426 (2021) 131316.
40. Pagliero M., Khayet M., Garcia-Payo C., Garcia-Fernandez L.; Hollow fibre polymeric membranes for desalination by membrane distillation technology: A review of different morphological structures and key strategic improvements; Desalination, 516 (2021) 115235.
41. Contreras-Martinez J., Garcia-Payo C., Khayet M.; Electrospun nanostructured membrane engineering using reverse osmosis recycled modules: Membrane distillation application; Nanomaterials, 11 (2021) 1601.
42. Mannina G., Alliet M., Brepols C., Comas J., Harmand J., Heran M., Kalboussi N., Makinia J., Robles A., Reboucas T.F., Ni B.-J., Rodriguez-Roda I., Victoria Ruano M., Bertanza G., Smets I.; Integrated membrane bioreactors modelling: A review on new comprehensive modelling framework; Bioresource Technology, 329 (2021) 124828.
43. Sauchelli Toran M., D'Haese A., Rodriguez-Roda I., Gernjak W.; Fouling propensity of novel TFC membranes with different osmotic and hydraulic pressure driving forces; Water Research, 17515 (2020) 115657.
44. Brepols C., Comas J., Harmand J., Heran M., Robles A., Rodriguez-Roda I., Ruano M.V., Smets I., Mannina G.; Position paper - progress towards standards in integrated (aerobic) MBR modelling; Water Science and Technology, 81 (2020) 1-91.
45. Antico, E.; Fontas, C.; Vera, R.; Mostazo, G.; Salvado, V; Guasch, H. A novel Cyphos IL 104-based polymer inclusion membrane (PIM) probe to mimic biofilm zinc accumulation. Science of the Total Environment 715 (2020) 136938.
46. Elias, G., Marguí, E., Díez, S., Fontàs, C. Polymer Inclusion Membrane as an Effective Sorbent to Facilitate Mercury Storage and Detection by X-ray Fluorescence in Natural Waters, Analytical Chemistry 90(7) (2018) 4756 -4763
47. Vera, R., Gelde, L., Anticó, E., Martínez de Yuso, M.V., Benavente, J., Fontàs, C. Tuning physicochemical, electrochemical and transport characteristics of polymer inclusion membrane by varying the counter-anion of the ionic liquid Aliquat 336, Journal of Membrane Science 529 (2017) 87-94.
48. Almecija M.C., Guadix A., Calvo J.I., Guadix E.M.; Changes in structure and performance during diafiltration of binary protein solutions due to repeated cycles of fouling/alkaline cleaning; Food and Bioproducts Processing, 105 (2017) 117 – 128.
49. Espejo-Carpio, F.J., Pérez-Gálvez, R., Almécija, M.D.C., Guadix, A., Guadix, E.M., Increasing the angiotensin converting enzyme inhibitory activity of goat milk hydrolysates by cross-flow filtration through ceramic membranes. Desalination and Water Treatment 56 (2015) 3544–3553.
50. Pérez-Gálvez, R., Guadix, E.M., Bergé, J.-P., Guadix, A., Processing fish press waters using metallic and ceramic filtration. Journal of Chemical Technology and Biotechnology 88 (2013) 1885–1890.
51. Ochando-Pulido J.M., Corpas-Martinez J.R., Vellido-Perez J.A., Martinez-Ferez A.; Optimization of polymeric nanofiltration performance for olive-oil-washing wastewater phenols recovery and reclamation; Separation and Purification Technology, 236 (2020) 116261.
52. Camacho M.A.N., Lopez A.I.G., Martinez-Ferez A., Ochando-Pulido J.M.; Two-phase olive-oil washing wastewater treatment plus phenolic fraction recovery by novel ion exchange resins process modelling and optimization; Separation and Purification Technology, 269 (2021) 118755.

53. Camacho M.A.N., Lopez A.I.G., Martinez-Ferez A., Ochando-Pulido J.M.; Increasing large-scale feasibility of two-phase olive-oil washing wastewater treatment and phenolic fraction recovery with novel ion exchange resins; Chemical Engineering and Processing - Process Intensification, 164 (2021) 108416,
54. Ruiz-Garcia A., Nuez I.; Performance evaluation and boron rejection in a SWRO system under variable operating conditions; Computers and Chemical Engineering, 153 (2021) 107441.
55. Ruiz-Garcia A., Nuez I., Carrascosa-Chisvert M.D., Santana J.J.; Simulations of BWRO systems under different feedwater characteristics. Analysis of operation windows and optimal operating points; Desalination, 491 (2020) 114582.
56. Ruiz-Garcia A., Nuez I.; Long-term intermittent operation of a full-scale BWRO desalination plant; Desalination, 489 (2020) 114526.
57. Lima-Rodriguez, A., Gonzalez-Herrera, A., Garcia-Manrique, J., Study of the dynamic behaviour of circular membranes with low tension, Applied Sciences, 9(21) (2019) 4716.
58. Gonzalez-Herrera, A., Garcia-Manrique, J., Numerical study of the mechano-acoustic coupled resonance of a tube-membrane system, Meccanica, 53 (2018) 3189–3207.
59. Gonzalez-Herrera, A., Olson, E.S., A study of sound transmission in an abstract middle ear using physical and finite element models. J. Acoust. Soc. Am. 138 (5) (2015) 2972–2985.
60. Hidalgo A.M., Leon G., Murcia M.D., Gomez M., Gomez E., Gomez J.L.; Using pressure-driven membrane processes to remove emerging pollutants from aqueous solutions; International Journal of Environmental Research and Public Health, 18 (2021) 4036.
61. Hidalgo A.M., Leon G., Gomez M., Murcia M.D., Gomez E., Macario J.A.; Removal of different dye solutions: A comparison study using a polyamide NF membrane; Membranes, 10 (2020) 408.
62. Sanchez-Moya, T., Hidalgo, A.M., Ros-Berruezo, G., Lopez-Nicolas, R..; Screening ultrafiltration membranes to separate lactose and protein from sheep whey: application of simplified model, Journal of Food Science and Technology, 57(9) (2020) 3193–3200
63. Alvarez J.R., Antón F.E., Alvarez-Garcia S., Luque S.; Treatment of aqueous effluents from steel manufacturing with high thiocyanate concentration by reverse osmosis; Membranes, 10 (2020) 437.
64. Anton E., Alvarez J.R., Palacio L., Prádanos P., Hernández A., Pihlajamaki A., Luque S.; Ageing of polyethersulfone ultrafiltration membranes under long-term exposures to alkaline and acidic cleaning solutions; Chemical Engineering Science, 134 (2015) 178-195.
65. Piedra E., Alvarez J.R., Luque S.; Hexavalent chromium removal from chromium plating rinsing water with membrane technology; Desalination and Water Treatment, 53 (2015) 1431-1439.
66. Reizabal A., Brito-Pereira R., Fernandes M.M., Castro N., Correia V., Ribeiro C., Costa C.M., Perez L., Vilas J.L., Lanceros-Mendez S.; Silk fibroin magnetoactive nanocomposite films and membranes for dynamic bone tissue engineering strategies; Materialia, 12 (2020) 100709.
67. Salazar H., Martins P.M., Santos B., Fernandes M.M., Reizabal A., Sebastian V., Botelho G., Tavares C.J., Vilas-Vilela J.L., Lanceros-Mendez S.; Photocatalytic and antimicrobial multifunctional nanocomposite membranes for emerging pollutants water treatment applications; Chemosphere, 250 (2020) 126299.
68. Mendes-Felipe C., Barbosa J.C., Goncalves R., Miranda D., Costa C.M., Vilas-Vilela J.L., Lanceros-Mendez S.; Lithium bis(trifluoromethanesulfonyl)imide blended in polyurethane acrylate photocurable solid polymer electrolytes for lithium-ion batteries; Journal of Energy Chemistry, 62 (2021) 485-496.
69. Hidalgo A.M., Leon G., Murcia M.D., Gomez M., Gomez E., Gomez J.L.; Using pressure-driven membrane processes to remove emerging pollutants from aqueous solutions; International Journal of Environmental Research and Public Health, 18 (2021) 4036.
70. Leon G., Hidalgo A.M., Miguel B., Guzman M.A.; Pertraction of CO(II) through novel ultrasound prepared supported liquid membranes containing D2EHPA. optimization and transport parameters; Membranes, 10 (2020) 436.
71. Leon G., Hidalgo A.M., Murcia M.D., Miguel B., Gomez E.; Determining kinetic constants and transport efficiencies at membrane interfaces to optimize the removal/recovery of Cu(II) through lulk liquid membranes containing benzoylacetone as carrier; Polish Journal of Environmental Studies, 26 (2017) 2879–2884.
72. Kaade, W., Güell, C., Ballon, A., Mellado-Carretero, J., De Lamo-Castellví, S., Ferrando, M. Dynamic membranes of tunable pore size for lemon oil encapsulation, LWT, 123 (2020) 109090.
73. Wang, J., Martínez-Hernández, A., de Lamo-Castellví, S., Romero, M.-P., Kaade, W., Ferrando, M., Güell, C. Low-energy membrane-based processes to concentrate and encapsulate polyphenols from carob pulp, Journal of Food Engineering, 281 (2020) 109996.
74. Rudolph, G., Virtanen, T., Ferrando, M., Güell, C., Lipnizki, F., Kallioinen, M. A review of in situ real-time monitoring techniques for membrane fouling in the biotechnology, biorefinery and food sectors, Journal of Membrane Science, 588 (2019) 117221.
75. Salamanca M., Lopez-Serna R., Palacio L., Hernandez A., Pradanos P., Pena M.; Study of the rejection of contaminants of emerging concern by a biomimetic aquaporin hollow fiber forward osmosis membrane; Journal of Water Process Engineering, 40 (2021) 101914.

76. Soto C., Torres-Cuevas E.S., Gonzalez-Ortega A., Palacio L., Lozano A.E., Freeman B.D., Pradanos P., Hernandez A.; Gas separation by mixed matrix membranes with porous organic polymer inclusions within o-hydroxypolyamides containing m-terphenyl moieties; Polymers, 13 (2021) 931.
77. Tanis-Kanbur M.B., Peinador R.I., Calvo J.I., Hernandez A., Chew J.W.; Porosimetric membrane characterization techniques: A review; Journal of Membrane Science, 619 (2021) 118750.
78. Hernandez-Suarez A., Rizo-Gorrita M., Suarez-Vega D., Velazco G., Gelfenstein I.R., Vazquez-Pachon C., Serrera-Figallo M.-A., Torres-Lagares D.; Effectiveness of silicon platelet-rich fibrin and autologous bone on bone regeneration in rabbit calvarian defects: A radiological and histological study; Applied Sciences, 11 (2021) 4074.
79. Toledano M., Gutierrez-Perez J.L., Gutierrez-Corrales A., Serrera-Figallo M.A., Toledano-Osorio M., Rosales-Leal J.I., Aguilar M., Osorio R., Torres-Lagares D.; Novel non-resorbable polymeric-nanostructured scaffolds for guided bone regeneration; Clinical Oral Investigations, 24 (2020) 2037-2049.
80. Toledano M., Toledano-Osorio M., Osorio R., Carrasco-Carmona A., Gutierrez-Perez J.-L., Gutierrez-Corrales A., Serrera-Figallo M.-A., Lynch C.D., Torres-Lagares D.; Doxycycline and zinc loaded silica-nanofibrous polymers as biomaterials for bone regeneration; Polymers, 12 (2020) 1201.
81. Julian I., Herguido J., Menendez M.; Gas permeation effect on the Two-Section Two-Zone Fluidized Bed Membrane Reactor (TS-TZFBMR) fluid dynamics: A CFD simulation study; Chemical Engineering Journal, 305 (2016) 201-211.
82. Esteras-Saz J., de la Iglesia O., Pena C., Escudero A., Tellez C., Coronas J.; Theoretical and practical approach to the dealcoholization of water-ethanol mixtures and red wine by osmotic distillation; Separation and Purification Technology, 270 (2021) 118793,
83. Martinez-Izquierdo L., Malankowska M., Tellez C., Coronas J.; Phase inversion method for the preparation of Pebax® 3533 thin film membranes for CO_2/N_2 separation; Journal of Environmental Chemical Engineering, 9 (2021) 105624.
